# A global 1 km resolution daily surface longwave radiation product from MODIS satellite data from 2000–2023

**DOI:** 10.1038/s41597-025-05076-8

**Published:** 2025-05-03

**Authors:** Jie Cheng, Qi Zeng, Hao Sun, Guo Yamin, Feng Yang, Mengfei Guo, Chenze Wu

**Affiliations:** 1 State Key Laboratory of Remote Sensing and Digital Earth, Faculty of Geographical Science, Beijing Normal University, Beijing, 100875 China; 2 Institute of Remote Sensing Science and Engineering, Faculty of Geographical Science, Beijing Normal University, Beijing, 100875 China

**Keywords:** Climate sciences, Atmospheric science

## Abstract

Surface longwave radiation (SLWR) is one of the two components of the surface radiation budget (SRB), which is a driving force of many land surface process models, the water cycle and climate change. To our knowledge, there are currently no publicly accessible long-term high-resolution (1 km) global daily surface longwave radiation products. We developed hybrid methods for estimating the 1 km resolution instantaneous clear-sky surface longwave upwelling radiation (SLUR) and surface longwave downwelling radiation (SLDR) and the cloud base temperature-based (CBT) single-layer cloud model (SLCM) for estimating the instantaneous cloudy-sky SLDR from MODIS data. The Essential thermaL Infrared remoTe sEnsing (ELITE) broadband emissivity (BBE) product and reanalysis surface temperature were employed to calculate the cloudy-sky SLUR. Synchronized with the diurnal information extracted from the ERA5 reanalysis data, we generated a 1 km resolution all-sky daily surface longwave radiation product (including SLUR and SLDR) via temporal integration. The produced daily SLUR and SLDR were validated via ground measurements collected from 369 sites distributed worldwide from nine flux networks. The root mean square error (RMSE) of the daily SLUR is less than 18 W/m^2^, and the RMSE of the daily SLDR is approximately 25 W/m^2^. The accuracy is commensurate with that of CERES-SYN.

## Background & Summary

As the driving force of many land surface processes, the surface radiation budget or net radiation (${R}_{n}$) plays a vital role in climate change and earth system science^1–3^. It is composed of surface shortwave net radiation (${R}_{ns}$) and longwave net radiation (${R}_{nl}$):

1 $$\begin{array}{c}{R}_{n}={R}_{ns}+{R}_{nl}\\ {R}_{nl}={R}_{L}\downarrow -{R}_{L}\uparrow ={\varepsilon }_{bb}{R}_{L}\downarrow -{\varepsilon }_{bb}\sigma {T}_{s}^{4}\end{array}$$

where *R*_*L*_↓ represents surface longwave downward radiation (SLDR), ${R}_{L}\uparrow$ represents surface longwave upward radiation (SLUR), ${\varepsilon }_{bb}$ represents surface broadband emissivity (BBE, 8–13.5 μm)^4^, $\sigma$ represents the Boltzmann constant, and ${T}_{s}$ represents the surface temperature.

There are usually three ways to obtain surface longwave radiation (SLWR) data^5–9^. The first is ground measurement, the most accurate method of obtaining the SLDR and SLUR via the so-called Pyranometer^10^. However, the spatial distribution of these sites is uneven, and the number of sites that we can access thus far is limited to approximately 500 worldwide. The land surface model (LSM) and general circulation model (GCM) can output spatially continuous coarse-resolution SLWR data. However, significant differences appeared among the simulated surface longwave components, as reported by Wild^11^. Satellite remote sensing is the only means of obtaining highly accurate regional and global SLWR data. During the past decades, a number of versatile methods or algorithms have been proposed to retrieve SLWR from satellite observations. Accordingly, several coarse-resolution (~1°) SLWR products have been generated, including the classical International Satellite Cloud Climatology Project-Flux Data (ISCCP-FD)^12^, the Global Energy and Water Cycle Experiment-Surface Radiation Budget (GEWEX-SRB)^13,14^, the Clouds and the Earth’s Radiant Energy System-Gridded Radiative Fluxes and Clouds (CERES-FSW)^15–17^, and the Satellite Application Facility on Climate Monitoring (CMSAF) surface longwave radiation products^18,19^. High spatial-resolution SLWR can be used to drive high-resolution LSMs and is extremely useful in climate modeling. unfortunately, such data are rarely scarce. To our knowledge, the long-time series 1 km global daily SLWR product that has been freely to the public is not available now.

The purpose of this study is to develop a global gridded high-resolution (1 km) daily SLWR product facilitating the study of climate monitoring and the Earth’s surface hydrological, ecological, and biogeochemical processes. These data were included in the EssentiaL thermaL Infrared remoTe sEnsing (ELITE) product suite (https://elite.bnu.edu.cn).

## Methods

**Fig. 1:**
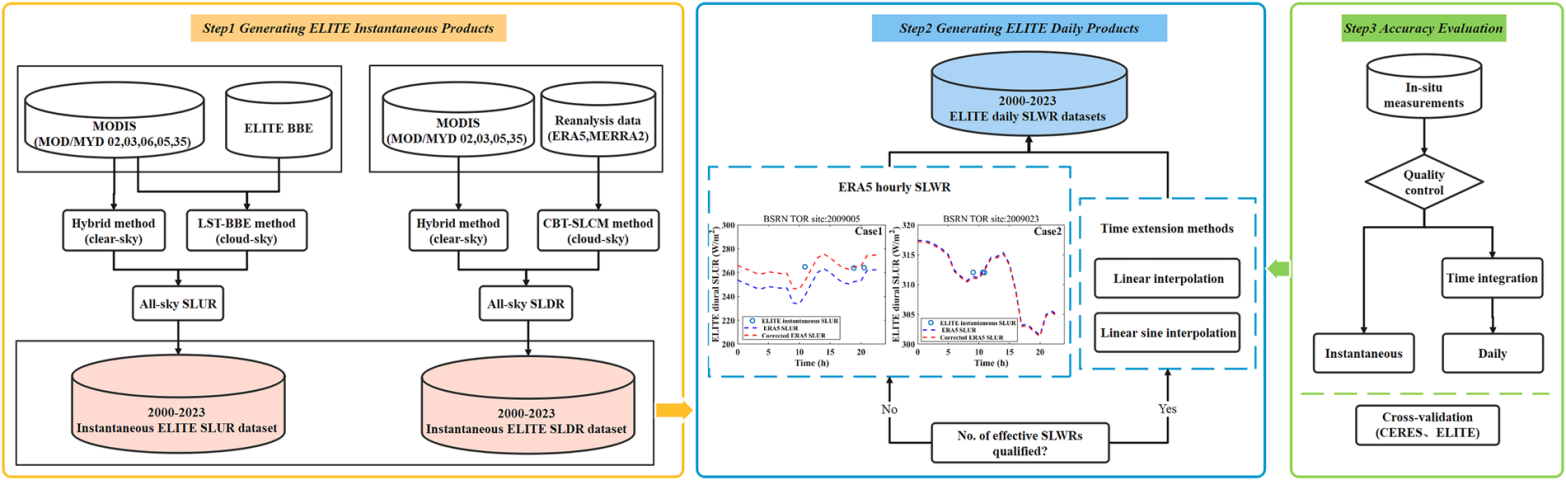
The flowchart of generating and evaluating the ELITE-MODIS daily SLWR product.

Figure 1 displays the flowchart used to generate the 1 km global daily SLWR data. The first step is estimating the instantaneous SLUR and SLDR under clear-sky and cloudy-sky conditions. Then, the estimated instantaneous SLUR and SLDR were projected to the sinusoidal projection to facilitate the daily SLUR and SLDR estimates. Finally, we generated the daily SLUR and SLDR via the time extension method in conjunction with the time change information extracted from the reanalysis ERA5 SLWR data. Both the estimated instantaneous and daily SLWRs were validated by *in situ* measurements, the latter was also evaluated by comparing with CERES SLWR product.

### Satellite data and auxiliary data

The MODIS level-1B and level-2 swath products from two polar-orbiting sun-synchronous satellites, Terra and Aqua (10:30 AM and 1:30 PM local solar time), were used to generate the instantaneous SLUR and SLDR products. These products are MOD/MYD02, MOD/MYD03, MOD/MYD35, MOD/MYD05, and MOD/MYD06. Specifically, MOD/MYD02 provides top-of-atmosphere (TOA) radiance for channels 29, 31, and 32, which are used to estimate the instantaneous clear-sky SLUR and SLDR. MOD/MYD03 was used for pixel geographical matching. MOD/MYD35 was utilized to identify cloud types. MOD/MYD05 provides the total water vapor content (CWV) with a spatial resolution of 1 km during the day and 5 km at night. The nighttime data are resampled to 1 km for consistency via the nearest neighbor interpolation method. MOD/MYD06 provides surface temperature, cloud top temperature, cloud optical thickness, cloud effective emissivity, and the cloud effective radius, which are used to estimate the cloudy-sky SLDR. Their spatial resolution was 5 km, and they were resampled to 1 km via the nearest neighbor method.

MERRA2 provides the atmospheric temperature profile, dew point temperature, and 2-meter air temperature, which are used to calculate the MODIS cloud base temperature (CBT), cloudy-sky atmospheric effective emissivity, and cloudy-sky SLDR. The CERES-SYN dataset offers daily SLWR data, which were used to cross-validate our daily SLWR product. The detailed data utilized are shown in Table 1.

**Table 1:** Detailed information on the employed satellite and reanalysis datasets.

Data source	Product name	Spatial resolution	Temporal resolution	Period	Coverage	Usage
MODIS	MOD/MYD 02	1 km	Instantaneous	2000–2023	Global	Clear-sky SLUR estimate
	MOD/MYD 03	1 km		2000–2023	Global	Pixels matching
	MOD/MYD 35	1 km		2000–2023	Global	Identifying cloud types
	MOD/MYD 05	1 or 5 km		2000–2023	Global	Clear-sky SLDR estimate
	MOD/MYD 06	5 km		2000–2023	Global	Cloudy-sky SLDR estimate Cloudy-sky SLUR estimate
MERRA2	inst6_3d_ana_Np	0.5° × 0.625°	6-hour	2000–2023	Global	Cloudy-sky SLDR estimate
	statD_2d_slv_Nx	0.5° × 0.625°		2000–2023	Global	Cloudy-sky SLDR estimate
ERA5	SLDR	25 km	Hourly	2000–2023	Global	Providing temporal information
	SLNR	25 km		2000–2023	Global	
CERES-SYN	DSLDR	100 km	Daily	/	Global	Cross-validation
	DSLUR	100 km		/	Global	

**Fig. 2:**
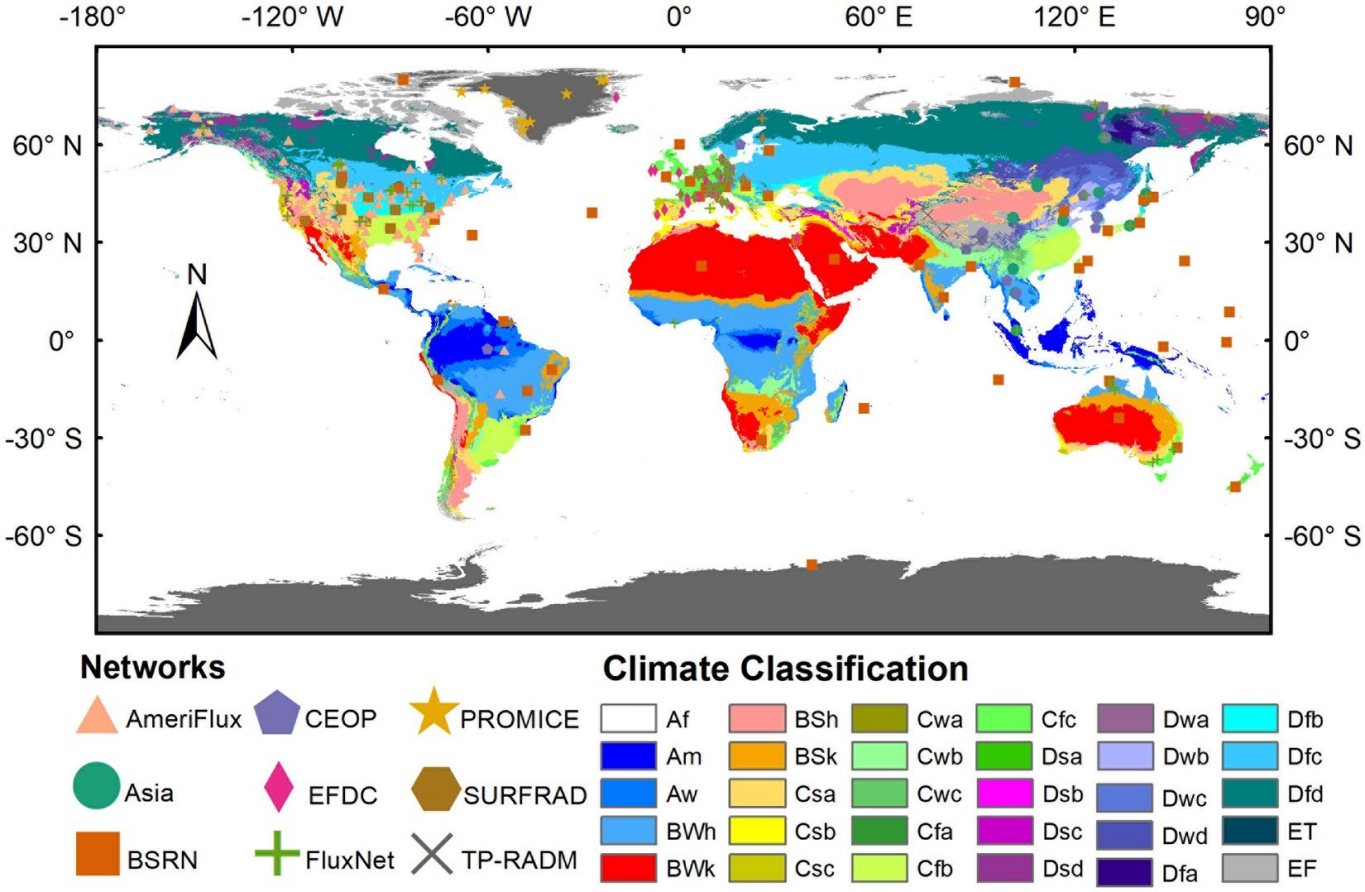
The geographical distribution of the *in situ* sites from nine flux networks. The full name of the Köppen climate classification category are: tropical rainforest climate (Af), tropical monsoon climate (Am), tropical savanna climate (Aw), tropical hot desert climate (Bwh), cold arid desert climate (Bwk), hot semi-arid climate (Bsh), hot semi-arid climate (Bsk), hot semi-arid climate (Bsk), hot summer mediterranean climate (Csa), cool summer mediterranean climate (Csb), subtropical monsoon climate (Cwa), temperate monsoon climate with dry winters climate (Cwb), humid subtropical climate (Cfa), oceanic climate (Cfb), mediterranean-influenced warm-summer humid continental climate (Dsb), subpolar oceanic climate (Dsc), humid continental climate with hot summers and dry winters (Dwa), warm-summer humid continental climate with dry winters (Dwb), subarctic monsoon climate (Dwc), humid continental climate with hot summers (Dfa), warm-summer humid continental climate (Dfb), warm-summer humid continental climate (Dfc), extremely cold subarctic climate (Dfd), tundra climate (Et) and snow and ice climate (Ef).

*In situ* data were employed to evaluate the accuracy of the ELITE SLWR product. We collected ground measurements at 369 sites from nine flux networks. Table 2 lists detailed information on the nine flux networks, including AmeriFlux^20^, the Asian network of flux sites (AsiaFlux)^20^, the Baseline Surface Radiation Network (BSRN)^21^, the Coordinated Energy and Water Cycle Observation Project (CEOP)^22^, the European Fluxes Database Cluster (EFDC)^23^, FluxNet^20,24^, the Programme for Monitoring of the Greenland Ice Sheet (PROMICE)^25^, the Surface Radiation Budget Monitoring (SURFRAD)^26^ and the integrated land‒atmosphere interaction observations on the Tibetan Plateau (TP-RADM)^27^. The spatial distribution of these sites is shown in Fig. 2. Most sites are located at mid-to-low latitudes, and the sites at high latitudes (>60°) are relatively sparse, especially in the Northern Hemisphere.

**Table 2:** Information regarding the nine flux networks.

Network	No. of sites	Observation frequency (min)	Website
AmeriFlux	105	30	https://ameriflux.lbl.gov/,
AsiaFlux	15	30	http://asiaflux.net/
BSRN	61	1	https://bsrn.awi.de/
CEOP	19	30	https://www.eol.ucar.edu/field_projects/ceop
EFDC	60	60	http://www.europe-fluxdata.eu/
FluxNet	84	30	https://fluxnet.fluxdata.org/
PROMICE	13	60	http://www.promice.org/
SURFRAD	7	1	https://gml.noaa.gov/grad/surfrad/index.html
TP-RADM	5	10	http://data.tpdc.ac.cn

^*^BSRN provides SLUR observations for only nine sites.

### Instantaneous SLUR estimate

The instantaneous 1 km clear-sky SLUR was retrieved via the hybrid method developed by Cheng *et al*.^28^, in which the linear relationship between the clear-sky SLUR and MODIS TOA thermal-infrared radiance of channels 29, 31, and 32 was established via extensive radiative transfer modeling. To ensure the applicability of the developed hybrid method globally, the temperature difference between the surface temperature and the bottom layer atmosphere temperature was incorporated during the development of the hybrid method. The surface was divided into three regions according to latitude: a low-latitude region (0°–30°N, 0°–30°S), a midlatitude region (30°–60°N, 30°–60°S), and a high-latitude region (60°–90°N, 60°–90°S). The temperature difference was statistically calculated from two years of the atmospheric infrared sounding (AIRS) L2 product and incorporated into radiative transfer modeling to guide the setting of surface temperature. The clear-sky SLUR is expressed as

2 $${{\rm{SLUR}}}_{{inst},\theta ,{lat}}={a}_{0,({inst},\theta ,{lat})}+{a}_{1,({inst},\theta ,{lat})}{L}_{29}+{a}_{2,({inst},\theta ,{lat})}{L}_{31}+{a}_{3,({inst},\theta ,{lat})}{L}_{32}$$

where ${{\rm{SLUR}}}_{{inst},\theta ,{lat}}$ is the instantaneous SLUR under different MODIS satellite view zenith angles (${VZA},\theta$) and latitudes; a_0_~a_3_ are the corresponding regression coefficients; and L_29_, L_31_, and L_32_ are the TOA radiances for MODIS channels 29, 31, and 32, respectively. According to Zeng *et al*.^29^, the bias and RMSE of the estimated instantaneous clear-sky SLUR were −4.33 and 18.15 W/m^2^ when validated at 141 flux sites from AmeriFlux, AsiaFlux, BSRN, CEOP, HiWATER-MUSOEXE, and TIPEX-III.

Under the cloudy-sky condition, the temperature–emissivity method was used to produce the instantaneous SLUR product, where the broadband emissivity (BBE, 8–13.5 μm) data were from the Essential thermaL Infrared remoTe sEnsing (ELITE) BBE product^4,30,31^ (https://elite.bnu.edu.cn), and the surface temperature was extracted from the MODIS06 product. Ultimately, we produced an all-sky instantaneous SLUR product.

#### Instantaneous SLDR estimate

The instantaneous 1 km clear-sky SLDR was retrieved via the hybrid method developed by Cheng *et al*.^32^. The idea of this hybrid method is inspired by the bulk formula^33^ and the study of Zhou *et al*.^34^. The estimated high-accuracy SLUR was employed as a proxy for the air temperature, and the TOA radiance of MODIS channel 29 was used to characterize the near-surface water vapor because its weighting function peaks at the surface. Following Zhou *et al*.^34^, total column water vapor (CWV) was also used as a predictor because water vapor is the major greenhouse gas in the atmosphere and has the largest spatial‒temporal variations over a short period of time. The clear-sky SLDR is expressed as

3 $${{SLDR}}_{{inst},{clr}}={a}_{0}+{a}_{1}{{SLUR}}_{{inst},{clr}}+{a}_{2}\log \left(1+{CWV}\right)+{a}_{3}\log {\left(1+{CWV}\right)}^{2}+{a}_{4}{L}_{29}$$

where ${{SLDR}}_{{inst},{clr}}$ is the instantaneous clear-sky SLDR, a_0_~a_4_ are the model coefficients, and ${{SLUR}}_{{inst},{clr}}$ is the instantaneous clear-sky SLUR derived by Eq. (2). The reported bias and RMSE of the estimated clear-sky SLDR are −3.77 and 26.94 W/m^2^, respectively, when validated at 141 flux sites from AmeriFlux, AsiaFlux, BSRN, CEOP, HiWATER-MUSOEXE, and TIPEX-III^29^.

Cloud-base temperature (CBT) is one of the key parameters that dominates the cloudy-sky SLDR; we estimate the 1 km cloudy-sky SLDR via the CBT-based single-layer cloud model (SLCM) developed by Yang and Cheng^35^. A global cloudy property database was constructed by combining the extracted cloud vertical structure (CVS) parameters from the active CloudSat data and the cloud properties from the passive MODIS data. Then, empirical methods for estimating the cloud geometrical thickness (CGT) under the ISCCP cloud classification system and the MODIS cloud classification system were developed separately. The CBT was calculated with the estimated CGT, MODIS cloud top height (CTH) and reanalysis atmospheric temperature profile. The cloudy-sky SLDR values were derived by feeding the estimated CBT and other parameters to the SLCM.

4 $${{SLDR}}_{{inst},{cld}}=\sigma {\varepsilon }_{a}{T}_{a}^{4}+\sigma {\varepsilon }_{c}{T}_{c}^{4}\times \left(1-{\varepsilon }_{a}\right)\times {cf}$$

where ${{SLDR}}_{{inst},{cld}}$ is the instantaneous cloudy-sky SLDR, ${\varepsilon }_{a}$ represents the atmospheric effective emissivity calculated by Prata’s formulation^36^, Ta is the near-surface air temperature, Tc is the cloudy base temperature and ${cf}$ is the cloud amount. The bias is better than −1.2 W/m^2^, and the RMSE values are less than 29.9 W/m^2^ when validated via *in situ* observations collected from the baseline surface radiation network (BSRN)^37^.

### Estimate of the daily SLWR

The daily averaged surface SLWR is required by agricultural, hydrological, and meteorological models, which can be derived by time integration of the instantaneous SLWR during one day. According to Zeng *et al*.^38^, the linear sine interpolation time extension method has good accuracy in estimating the daily SLWR. We adopted this method to calculate the daily SLWR data. However, this method requires a certain number of instantaneous SLWR values within one day. If the requirement was not met, we adjusted the ERA5 instantaneous values via the retrieved instantaneous data and then calculated the ELITE daily average from these corrected values. On the basis of the produced instantaneous SLWR product, we first converted it from swath format to tile format to facilitate the statistics of the number and timing of valid instantaneous SLWR values during one day. Typically, ELITE SLWR has at least four overpasses per day. Then, by combining linear sine interpolation and piecewise linear interpolation methods, we integrated and summed the instantaneous values into the daily mean SLWR. The specific formulas are provided in (4)-(6).

5 $${SLWR}\left(t\right)=a\times \sin \left[\frac{\left(t-{t}_{{sr}}\right)\pi }{{t}_{{ss}}-{t}_{{sr}}}\right]+{\overline{{SLWR}}}_{{night}}$$

6 $${SLWR}\left(t\right)=\frac{{t}_{2}-t}{{t}_{2}-{t}_{1}}{SLWR}\left({t}_{1}\right)+\frac{t-{t}_{1}}{{t}_{2}-{t}_{1}}{SLWR}\left({t}_{2}\right)$$

7 $${SL}{{WR}}_{{average}}^{{daily}}=\frac{{\int }_{0}^{{t}_{{sr}}}{\overline{{SLWR}}}_{{night}}{\rm{d}}t+{\int }_{{t}_{{sr}}}^{{t}_{{ss}}}S{LWR}\left(t\right){\rm{d}}t+{\int }_{{t}_{{ss}}}^{24}{\overline{{SLWR}}}_{{night}}{\rm{d}}t}{24}$$

where *t*_*sr*_ and *t*_*ss*_ are the sunrise and sunset times, respectively. where ${\overline{{SLWR}}}_{{night}}$ is the average SLWR value of the nighttime data, *a* is the amplitude calculated via least-squares fitting, and *t* is the time between *t*_*1*_ and *t*_*2*_, where *t*_*1*_ and *t*_*2*_ are the times between sunrise and sunset, respectively. The specific technical process of calculating daily SLWR values is as follows:

- There must be at least two daytime observations and one or more nighttime observations per day. If all the daytime SLWRs are much greater than the SLWRs during the entire night, linear sine interpolation is used to calculate the daily SLWR. Otherwise, the piecewise linear interpolation method was used. When the linear sine interpolation is used to calculate the daily SLWR, the amplitudes produced by the daytime SLWRs must be positive. If the condition were not met, piecewise linear interpolation was used.
- If the number of daytime observations is insufficient, we used the hourly ERA5 values adjusted by the retrieved instantaneous values on that day to calculate the ELITE daily mean, as shown in Fig. 1 (Case 1). The process consisted of two parts: (1) the differences between the ELITE instantaneous and the matched ERA5 SLWRs were calculated, and then these differences were averaged, i.e., the mean bias; (2) the mean bias was added to the ERA5 SLWRs on that day [1,24], and then the adjusted ERA5 SLWRs were used to calculate the ELITE daily SLWR via time integration.
  8 $$bia{s}_{inst}=mean(ELITE\,SLW{R}_{inst}-ERA5\,SLW{R}_{inst})$$
  9 $${SL}{{WR}}_{{average}}^{{daily}}=\frac{\mathop{\sum }\limits_{1}^{24}{ERA}5\,{{SLWR}}_{{inst}}}{24}+{{bias}}_{{inst}}$$
  where ${{bias}}_{{inst}}$ is the mean of the difference between the ELITE instantaneous SLWR and the ERA hourly SLWR.
- If the number of nighttime observations is insufficient, we use the hourly ERA5 values adjusted by the retrieved instantaneous values on that day to calculate the ELITE daily mean, as shown in Fig. 1 (Case 2).

Statistical metrics. The performance of the retrieved instantaneous and daily surface longwave radiation products was evaluated via bias, the root mean square error (RMSE) and the determination coefficient (R^2^). The formulae are expressed as follows:

10 $${\rm{Bias}}=\frac{1}{n}\mathop{\sum }\limits_{i=1}^{n}({SL}{{WR}}_{{pre}}-{SLW}{R}_{{obs}})$$

11 $${\rm{RMSE}}=\sqrt{\frac{1}{n}\mathop{\sum }\limits_{i=1}^{n}{({SL}{{WR}}_{{pre}}-{SL}{{WR}}_{{obs}})}^{2}}$$

12 $${R}^{2}=1-\frac{{\sum }_{i=1}^{n}{({SL}{{WR}}_{{obs}}-{SLW}{R}_{{pre}})}^{2}}{{\sum }_{i=1}^{n}{({SL}{{WR}}_{{obs}}-{\overline{{SLWR}}}_{{obs}})}^{2}}$$

where ${SLW}{R}_{{obs}}$ and ${SLW}{R}_{{pre}}$ represent the observed and retrieved values, respectively. ${\overline{{SLWR}}}_{{obs}}$ represents the means, and n represents the number of observations.

## Data Records

The ELITE daily SLWR product from 2000–2023 (continuously updated) is freely available from Zenodo^39,40^. The SLDR and SLUR data are released separately. The size of the SLDR product is approximately 278 gigabytes/year and 6.4 trillion bytes for 24 years. The size of the SLUR product is the same as that of the LSDR. They were stored in zip-compressed files, one file per day, named “YYYYDOY.zip”, where “YYYY” denotes the year and “DOY” denotes the Julian day of the year. The data were organized in the Hierarchical Data File Version 5 (HDF5) format containing the values of the SLDR and the quality check (QC) flags within each daily file and named “ELITE.Daily.SLDR.YYYYDOY_h**v**.h5”, where “ELITE” denotes the product name; “YYYY”, “DOY”, and “h**v**” denote the year, Julian day of the year, and row and column numbers of each tile, respectively. The daily SLDR values are provided in a sinusoidal projection and stored as 16-bit integer data types in units of W/m^2^. They are converted by subtracting an offset of 1000 and multiplying by a scale factor of 0.05, with an invalid value of 999. Data details are provided in Table 3. The QC flags are dimensionless and stored as a 16-bit unsigned integer data type, providing information regarding the sources and quality of the estimated SLDR. The detailed information is shown in Table 4.

**Table 3:** Detailed information about the scientific data sets (SDSs) in the ELITE SLWR product.

SDS Name	Long Name	Number Type	Unit	Reference range	Fill Value	Scale Factor	Added Offset
SLDR_daily	Daily surface longwave downward radiation	uint16	W/m^2^	[100, 500]	999	0.05	1000
SLDR_qc	Quality control for daily SLDR	uint16	/	/	999	/	/
SLUR_ daily	Daily surface longwave upward radiation	uint16	W/m^2^	[100, 600]	999	0.05	1000
SLUR_qc	Quality control for daily SLUR	uint16	/	/	999	/	/
Projs	projections	/	/	/	/	/	/
Geotrans	geotransformation	/	m	/	/	/	/

**Table 4:** Explanations of the QC flag in the ELITE daily SLWR product.

Digits	SDS Name	Long Name	Bit combination	Detailed information
0~1	alg_flag	algorithm flag	00	Invalid
			01	Linear sine interpolation
			10	Piecewise linear interpolation
			11	ERA5_Fill
2~3	polar_flag	Polar region processing method for algorithm	00	Normal
			01	Polar day
			10	Polar night
			11	Unused
4~7	night_valid_count	Night valid count for instantaneous SLWR	uint4 (0–15)	Number of valid instantaneous SLWR for night
8~11	day_valid_count	Day valid count for instantaneous SLWR	uint4 (0–15)	Number of valid instantaneous SLWR for day
11~15	Unused	/	/	/

The organization of SLUR data is the same as that of SLDR data. We did not delineate it to avoid redundancy.

## Technical Validation

### *In situ* validation

**Fig. 3:**
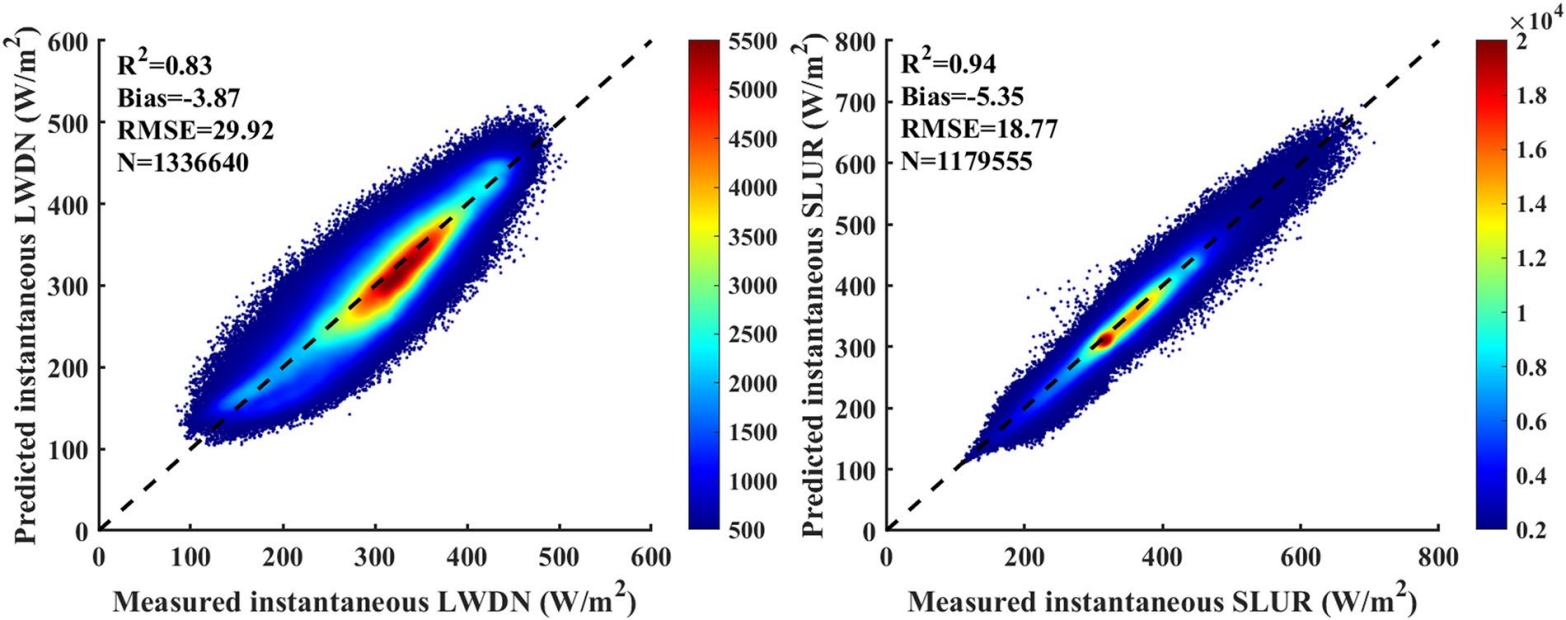
The scatter plots between the ELITE instantaneous SLWR and ground measurements.

**Fig. 4:**
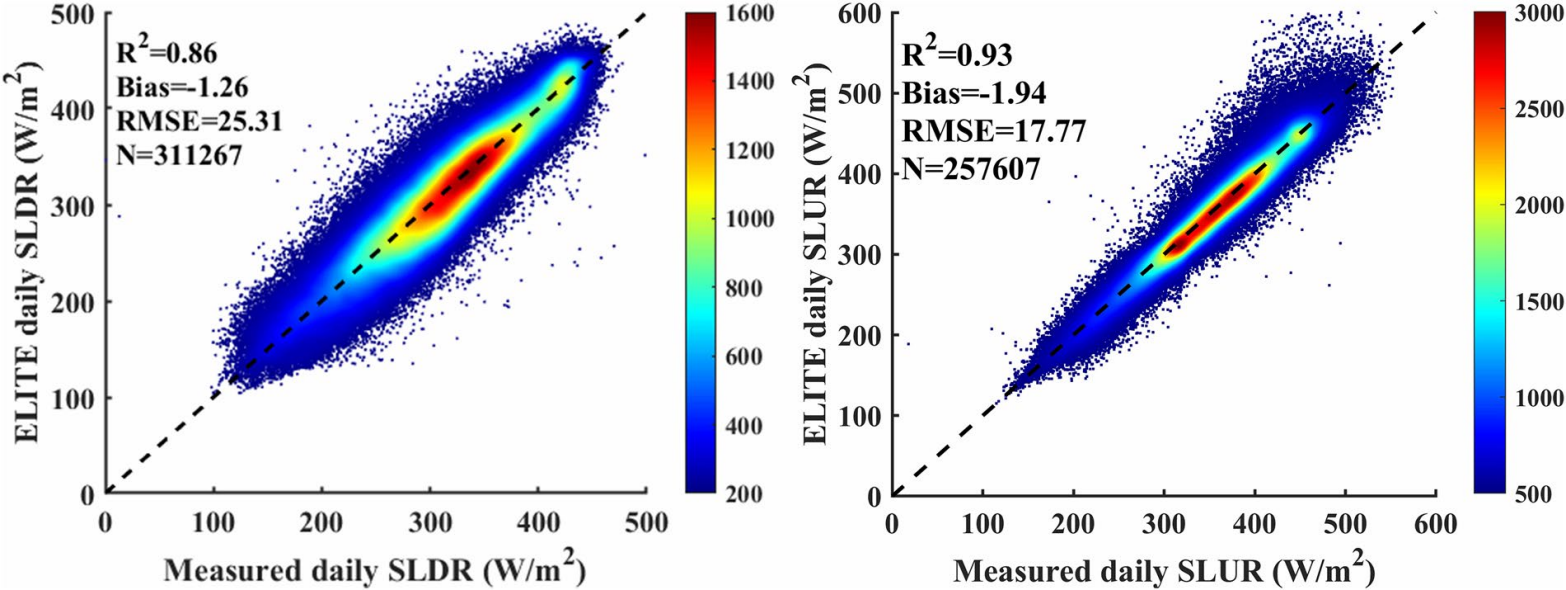
The scatter plot between the daily ELITE SLWR and ground-measured SLWR.

We validated the instantaneous SLDR and SLUR via ground measurements collected from 369 flux sites. As shown in Fig. 3, *in situ* validation yields bias, RMSE and R^2^ of −3.87 W/m^2^, 29.92 W/m^2^ and 0.83, respectively, for SLDR and −5.35, 18.77 W/m^2^ and 0.94, respectively, for SLUR. Since the overall biases are acceptable, we directly apply the instantaneous SLDR and SLUR to calculate the daily SLDR and SLUR in the following. The validation results for the ELITE daily SLWR are shown in Fig. 4. The ELITE daily SLDR and SLUR agreed well with the ground-measured values. The bias, RMSE and R^2^ are −1.26 W/m^2^, 25.31 W/m^2^ and 0.86, respectively, for the SLDR. The values are −1.94 W/m^2^, 17.77 W/m^2^ and 0.93 for the daily SLUR.

**Fig. 5:**
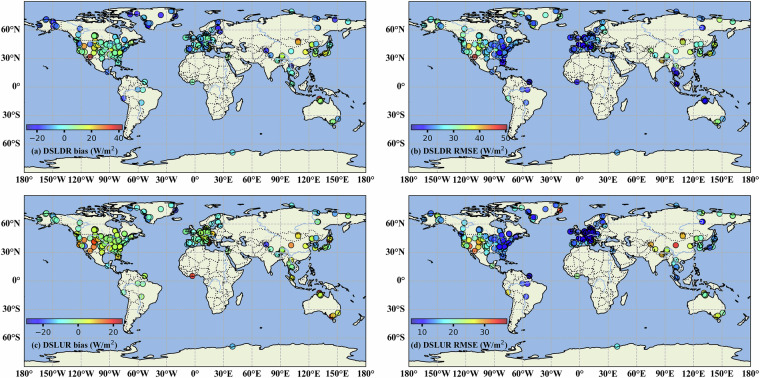
Spatial distribution of the bias and RMSE values of the ELITE daily SLDR (DSLDR) and SLUR (DSLUR) at flux sites.

The spatial performance of the ELITE daily SLWR product represented by the bias and RMSE at each *in situ* site is presented in Fig. 5. For daily SLDR, most sites have an RMSE in the range of 20–30 W/m^2^, with only 1~2 sites having an RMSE greater than 40 W/m^2^. Most biases are negative, with only a few sites having positive biases greater than 20 W/m^2^. With respect to the ELITE daily SLUR, the RMSE mainly ranges from 10 to 20 W/m^2^, with some sites exceeding 30 W/m^2^, and the biases are mostly approximately zero, but some sites have bias values of approximately 20 W/m^2^.

**Fig. 6:**
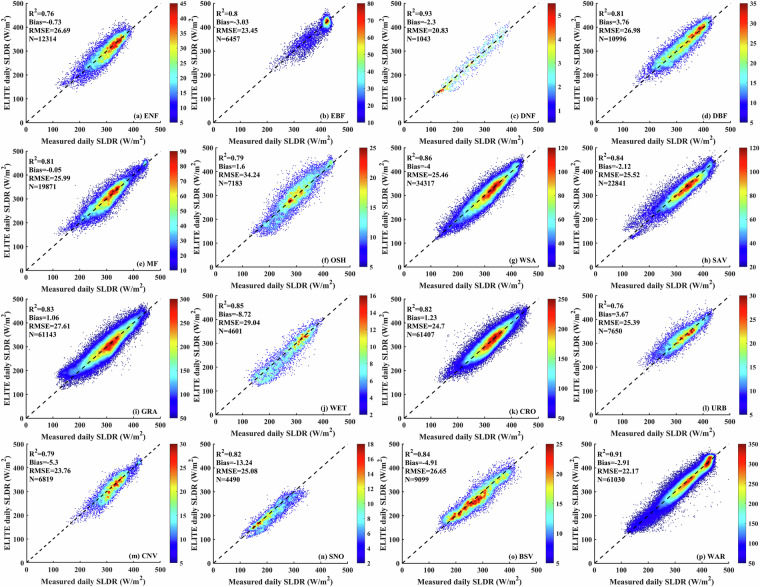
Overall performance of the ELITE daily SLDR for different land cover types. The full name of IGBP land cover types are: Evergreen needleleaf forests (ENF), Evergreen broadleaf forests (EBF), Deciduous needleleaf forests (DNF), Deciduous broadleaf forests (DBF), Mixed forests (MF), Closed shrublands (CSH), Open shrublands (OSH), Woody savannas (WSA), Savannas (SAV), Grasslands (GRA), Permanent wetlands (WET), Croplands (CRO), Urban and build-up lands (URB), Cropland/natural vegetation mosaics (CNV), Permanent snow and ice (SNO), Permanent snow and ice (SNO), Barren (BSV) and Water bodies (WAT).

**Fig. 7:**
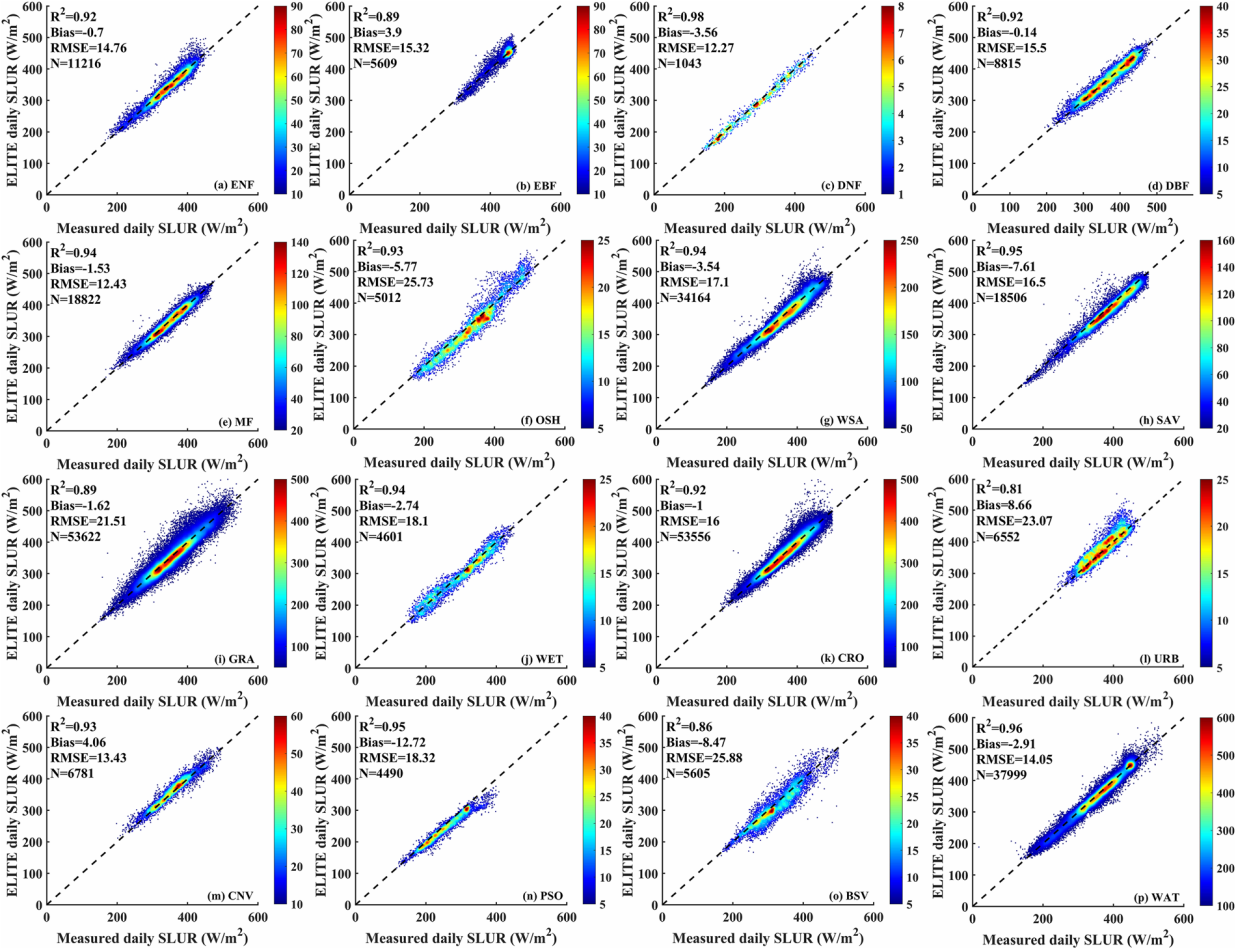
Overall performance of the ELITE daily SLUR for different land cover types.

The 369 collected *in situ* sites cover various surface types worldwide. These sites belong to 16 different surface types, according to the International Geosphere-Biosphere Programme (IGBP) land cover from MCDQ21 data (seventeen surface types), indicating that our collected sites can well represent global surface types. We evaluated the performance of the ELITE SLWR under different surface types. The accuracy statistics of the ELITE SLWR product are shown in Figs. 6, 7. For daily SLDR, excluding surface types with RMSEs greater than 29 W/m^2^, the RMSE is between 20.83 W/m^2^ and 26.98 W/m^2^, the minimum bias is −0.05 W/m^2,^ the maximum negative bias is −13.24 W/m^2^, and the average R^2^ is 0.83. The minimum RMSE of 20.83 W/m^2^ and the highest R^2^ of 0.98 are both achieved in deciduous needleleaf forests. Permanent snow and ice have the largest bias of −13.24 W/m^2^ because most sites are from the PROMICE network, with a bias of −11.29 W/m^2^ for instantaneous all-sky SLDRs, which is significantly greater than the overall global bias (shown in Fig. 3). The worst RMSE greater than 29 W/m^2^ is found for permanent wetlands and open shrublands, which may be caused by the significant spatial heterogeneity affecting the instantaneous SLDR along with insufficient effective instantaneous measurements impacting the estimate of daily average values.

The daily SLUR shows good performance as a whole. One-sixth of the surface types have RMSEs greater than 20 W/m^2^. The RMSEs of the remaining surface types are between 12.27 W/m^2^ and 18.32 W/m^2^, with a minimum bias of −0.14 W/m^2,^ a maximum bias of 8.66 W/m^2^, and an average R^2^ of 0.92. The minimum RMSE of 12.27 W/m^2^ and the highest R^2^ of 0.98 are also achieved in deciduous needleleaf forests, whereas deciduous broadleaf forests has the smallest bias of −0.14 W/m^2^. The worst RMSE around 25 W/m^2^ is found for open shrublands, urban and built-up lands, and barren land, which may be due to significant differences in radiation characteristics caused by high spatial heterogeneity, the urban heat island effect leading to temperature anomalies, and changes in bare soil and vegetation cover.

The accuracy of the ELITE daily SLWR product under different climate conditions was evaluated, and the results are summarized in Table 5. According to the Köppen climate classification (1991–2020)^41^, the collected sites are distributed across a total of 24 climate types worldwide. For ELITE daily SLDR, the minimum bias is −1.23 W/m^2^, the maximum bias is −10.88 W/m^2^, the RMSE generally ranges from 15.99 to 28.01 W/m^2^, and the average R² is 0.72 for most climate types. The minimum bias of −1.23 W/m^2^ is achieved in Dfb, while Am has the smallest RMSE of 15.99 W/m^2^, and Dfd has the highest R^2^ of 0.93. However, the ELITE daily SLDR performances were seriously underestimated and overestimated for specific climate types, including Bsk, Cwa, Cwb, Dsb, Dwa, and Dwb, with a maximum negative bias of −26.8 W/m2 in Cwa and a positive bias of 19.46 W/m^2^ in Bsk. The highest RMSE of 37.59 W/m^2^ is noted in Bsk, whereas the lowest R² of 0.22 occurs in Cwb. These differences may be due to the significant seasonal variations, extremes, and complexity of the cloud amount changes in these climate types. For example, cloud patterns frequently fluctuate in the Bsk climate type; Dsb and Dwb are strongly influenced by terrain, and Cwa may experience too great a cloud amount^42^. These factors likely contribute to inaccuracies in daily SLDR estimates.

**Table 5:** The accuracies of the ELITE daily SLWR product for the different climate types.

Climate Types	Daily SLDR			Daily SLUR		
	Bias (W/m^2^)	RMSE (W/m^2^)	R^2^	Bias (W/m^2^)	RMSE (W/m^2^)	R^2^
Af	−1.52	16.87	0.41	3.4	13.27	0.12
Am	−2.38	15.99	0.6	2.65	12	0.01
Aw	2.37	19.69	0.71	3.5	20.57	0.46
Bwh	−3.44	26.89	0.63	/	/	/
Bwk	−4.37	29.14	0.76	−15.06	28.65	0.86
Bsh	−6.05	24.48	0.75	−10.03	13.2	0.97
Bsk	19.46	37.59	0.73	3.87	27.13	0.86
Csa	−1.76	21.13	0.71	−2.61	18.79	0.82
Csb	−6.51	28.01	0.53	−0.33	20.05	0.83
Cwa	−26.8	32.32	0.74	6.73	19.02	0.53
Cwb	−17.03	29.95	0.22	−5.14	27.47	0.1
Cfa	3.27	24.91	0.82	−1.92	18.61	0.87
Cfb	−5.92	21.57	0.74	−6.36	12.54	0.92
Dsb	16.6	32.83	0.52	15.21	25.44	0.82
Dsc	−1.58	29.72	0.83	−1.76	21.25	0.93
Dwa	12.48	32.11	0.84	1.13	26.28	0.87
Dwb	1.95	26.18	0.83	−3.98	16.05	0.95
Dwc	12.79	37.12	0.69	5.5	33.37	0.72
Dfa	3.32	26.47	0.81	−1.37	16.38	0.93
Dfb	−1.23	25.65	0.79	0.14	14.18	0.93
Dfc	−8.97	27.51	0.81	−2.43	16.86	0.93
Dfd	−2.3	20.83	0.93	−3.56	12.27	0.98
Et	−5.04	29.39	0.75	−8.27	21.51	0.88
Ef	−10.88	25.21	0.74	−10.19	15.12	0.94

For the ELITE daily SLUR, the minimum bias is 0.14 W/m^2^, the maximum bias is −10.19 W/m^2^, the RMSE ranges from 12 W/m^2^ to 20.57 W/m^2^, and the average R² is 0.76 for most climate types. The minimum bias of 0.14 W/m^2^ is achieved in Dfb, while Am has the minimum RMSE of 12 W/m^2^, and Dfd has the highest R^2^ of 0.98. With respect to the Bwk, Cwb, Dsb, and Dwa climate types, the ELITE daily SLUR performances were seriously underestimated and overestimated, with a maximum negative bias of −15.06 W/m^2^ for Bwk and a positive bias of 15.21 W/m^2^ for Dsb. The RMSEs slightly exceed 25 W/m^2^. The reason may be that these climate types belong to extreme climatic conditions with significant temperature fluctuations and that there are generally only four instantaneous values available, which may make it difficult to capture their diurnal variations accurately.

### Cross validation

**Fig. 8:**
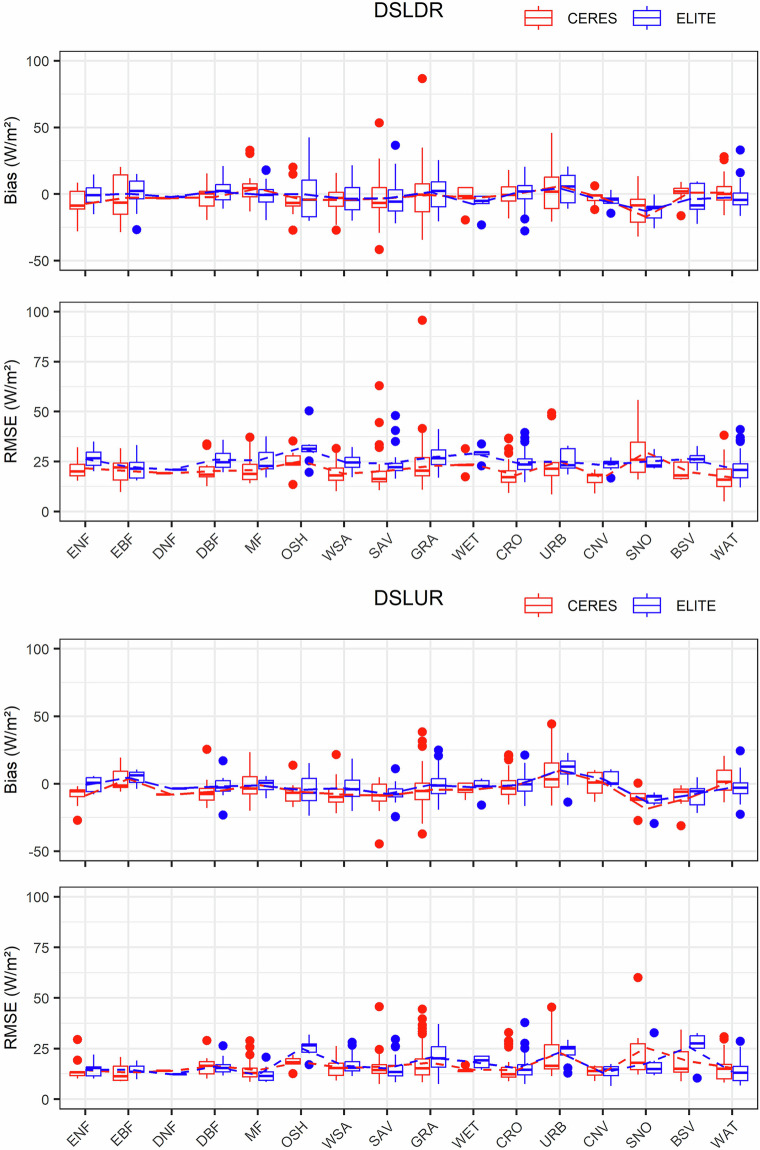
The validation results of the daily ELITE and CERES-SYN SLWR products under different surface types. The blue color represents ELITE and the red color represents CERES-SYN.

**Fig. 9:**
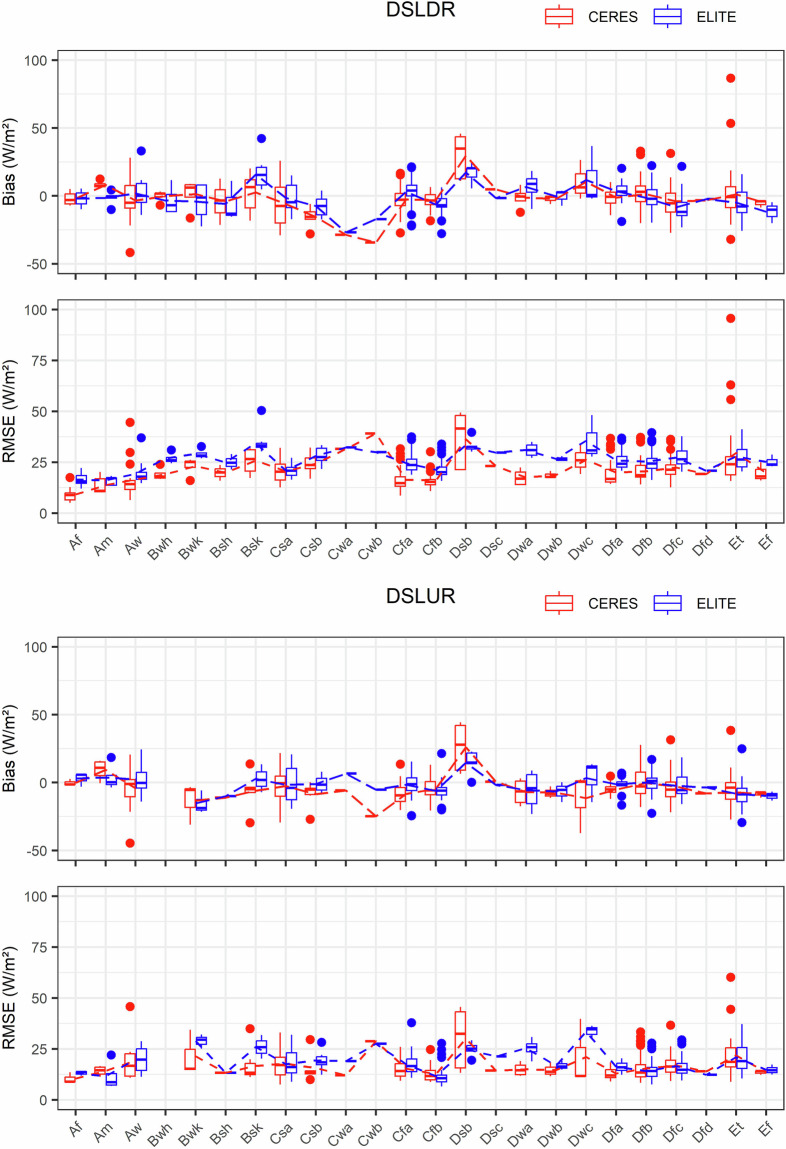
The validation results of the daily SLWR products from ELITE and CERES-SYN under different climate types. The red color represents CERES-SYN, and the blue color represents ELITE.

CERES-SYN is the only long-term satellite SLWR product that is freely available to the public. We compared the ELITE daily SLWR product with CERES-SYN under different surface types and climate types. The comparison results are shown in the boxplots in Figs. 8, 9. The absence of boxes for some surface types or climate types is due to insufficient *in situ* site numbers, but the lines can represent the average values of bias and RMSE. The solid dots represent anomalies in the accuracy for each surface type or climate type.

As shown in Fig. 8, the RMSEs of ELITE daily SLDR are slightly greater than those of CERES-SYN. The RMSE is approximately 25 W/m^2^ for the former and 22~25 W/m^2^ for the latter. Most of the biases of both SLDRs are close to zero. Both SLDR products have poor accuracy for certain surface types, such as open shrublands, savannas, and water bodies. On the basis of the outliers of *in situ* site accuracies across different surface types, the overall uncertainty of daily ELITE SLDR is smaller than that of CERES-SYN, indicating that the performance of the ELITE daily SLDR product is more stable than that of CERES-SYN for these surface types.

The RMSEs of ELITE daily SLUR and CERES-SYN SLUR are comparable under different surface types, with average RMSEs below 20 W/m^2^ and average biases of approximately zero. The first noticeable outlier appears in the CERES-SYN SLUR product, with the surface type of permanent snow and ice, where the RMSE exceeds 60 W/m^2^ and the bias exceeds 50 W/m^2^. Although ELITE SLUR also shows an outlier for this surface type, its RMSE is approximately 30 W/m^2^, and the bias is approximately −25 W/m^2^, which is lower than that of CERES-SYN. The surface types with outliers in the daily SLUR are similar to those in the daily SLDR. Overall, the performance of the ELITE daily SLUR product is also more stable than that of the CERES-SYN daily SLUR.

Figure 9 shows the accuracies of the ELITE and CERES-SYN daily SLWR products for different climate types. For the daily SLDR, the overall trends of bias and RMSE for ELITE and CERES-SYN are similar across different climate types. The ELITE SLDR shows slightly higher RMSEs (less than 25 W/m^2^) than those of CERES-SYN and mean biases closer to zero in most climate types, whereas the CERES-SYN SLDR has more outliers in certain climate types (e.g., Aw and Et), especially Et, indicating that its performance is less stable than that of the ELITE SLDR under these conditions.

There are no SLUR values for Bwh because none of the sites within this climate type provided SLUR measurements. The RMSEs of the ELITE daily SLUR under most climate types do not differ much from those of CERES-SYN, being below 20 W/m², but there are significant differences in the average RMSEs for Bwk, Bsk, Dwa, and Dwc. The biases of the ELITE SLUR are more concentrated around zero than those of CERES-SYN across different climate types. Compared with CERES-SYN, ELITE has fewer outliers and less deviation, indicating that its performance is more stable than that of CERES-SYN across different climate types.

Combining the evaluation results in Figs. 8, 9, the performance of the daily ELITE SLWR product is more stable than that of the CERES-SYN SLWR product. Note that the spatial resolution of ELITE and CERES-SYN SLWR products is different, the former is 1 km while the latter is 100 km. The spatial resolution difference is not considered during the comparison.

**Fig. 10:**
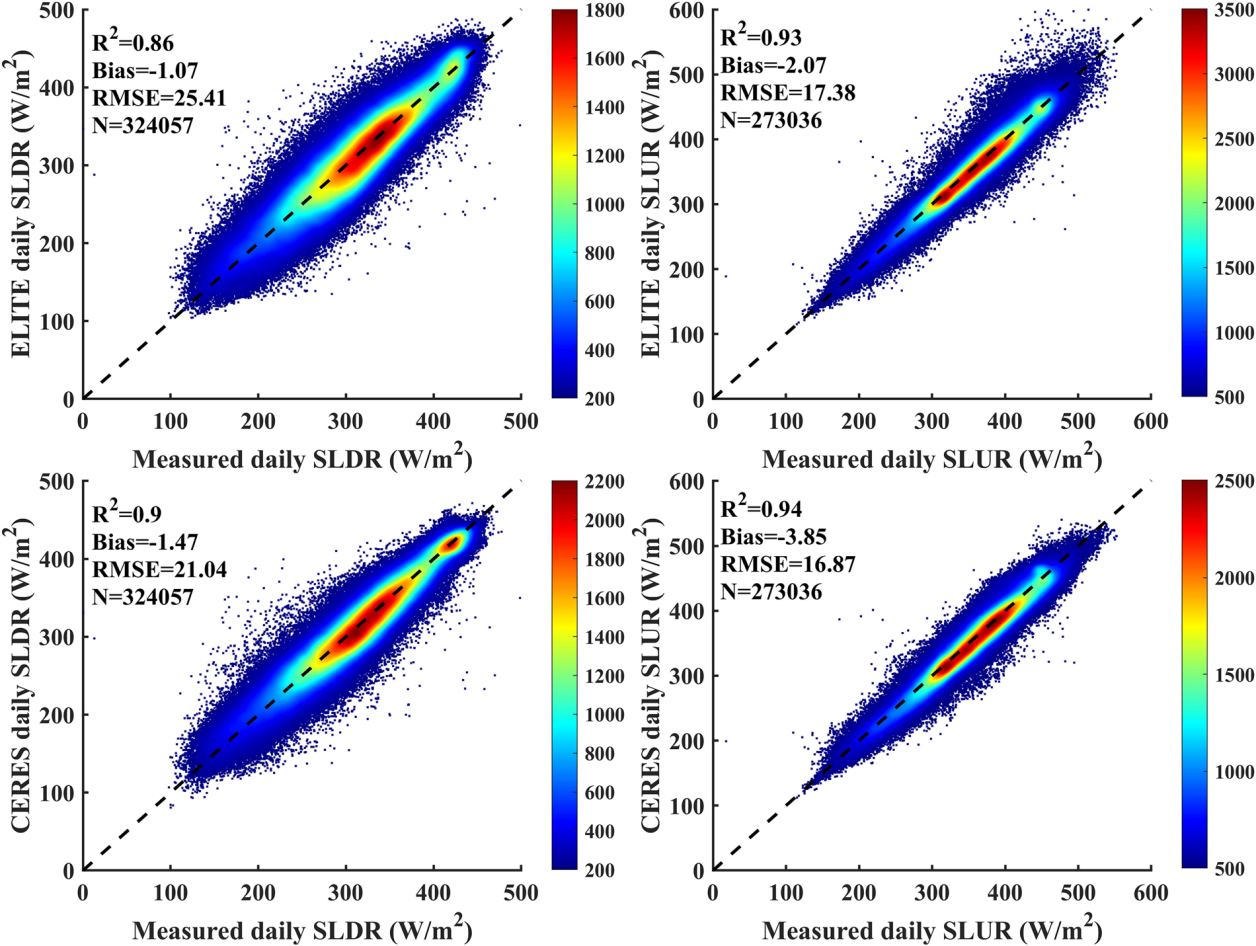
Overall accuracy of the daily ELITE and CERES-SYN SLWR products, respectively.

After the outliers were analyzed and the *in situ* site with the most significant impact on the accuracy of CERES-SYN was removed, we calculated the overall accuracies of the ELITE and CERES-SYN daily SLWR products. As shown in Fig. 10, the bias of the daily ELITE SLDR is −1.07 W/m^2^, and the bias of the CERES-SYN SLDR is relatively poor, with a value of −1.47 W/m^2^. The RMSE of the daily CERES-SYN SLDR is better than that of the ELITE SLDR; the values are 21.04 and 25.41 W/m^2^, respectively. The bias and RMSE of the daily ELITE SLUR are −2.07 and 17.38 W/m^2^, whereas the values are −3.85 and 16.87 W/m^2^ for CERES-SYN, respectively. The R^2^ values are both greater than 0.93. In conclusion, the accuracy of the daily ELTE SLWR is compensated with that of CERES-SYN.

## Usage Notes

In this study, we provide a global spatiotemporally continuous daily ELITE SLWR product from 2000 to 2023 for various applications and studies. The product was stored in hdf5 format as unsigned 16-bit with one file per day, and users can use MATLAB, Python, IDL, etc., to read and manipulate the data. Note that the data extracted from the SDSs must be multiplied by their corresponding scale factors (in Table 3). The uncertainties were greater than the normal reference range (Table 3) and should be used with caution. We update the product when new data sources become available.

## Data Availability

All the code used in this study for data processing and product generation is openly available at Zonodo (https://zenodo.org/records/14209566)^43^.
